# Improved prediction of long-term kidney outcomes in people with type 2 diabetes by levels of circulating haematopoietic stem/progenitor cells

**DOI:** 10.1007/s00125-023-06002-6

**Published:** 2023-09-15

**Authors:** Benedetta Maria Bonora, Mario Luca Morieri, Marella Marassi, Roberta Cappellari, Angelo Avogaro, Gian Paolo Fadini

**Affiliations:** 1 Department of Medicine, University of Padova, Padua, Italy; 2 Veneto Institute of Molecular Medicine, Padua, Italy

**Keywords:** End-stage kidney disease, Inflammation, Non-albuminuric, Regeneration, Stratification

## Abstract

**Aim/hypothesis:**

We examined whether prediction of long-term kidney outcomes in individuals with type 2 diabetes can be improved by measuring circulating levels of haematopoietic stem/progenitor cells (HSPCs), which are reduced in diabetes and are associated with cardiovascular risk.

**Methods:**

We included individuals with type 2 diabetes who had a baseline determination of circulating HSPCs in 2004–2019 at the diabetes centre of the University Hospital of Padua and divided them into two groups based on their median value per ml of blood. We collected updated data on eGFR and albuminuria up to December 2022. The primary endpoint was a composite of new-onset macroalbuminuria, sustained ≥40% eGFR decline, end-stage kidney disease or death from any cause. The analyses were adjusted for known predictors of kidney disease in the population with diabetes.

**Results:**

We analysed 342 participants (67.8% men) with a mean age of 65.6 years. Those with low HSPC counts (*n*=171) were significantly older and had a greater prevalence of hypertension, heart failure and nephropathy (45.0% vs 33.9%; *p*=0.036), as evidenced by lower eGFR and higher albuminuria at baseline. During a median follow-up of 6.7 years, participants with high vs low HSPC counts had lower rates of the composite kidney outcome (adjusted HR 0.69 [95% CI 0.49, 0.97]), slower decline in eGFR and a similar increase in albuminuria. Adding the HSPC information to the risk score of the CKD Prognosis Consortium significantly improved discrimination of individuals with future adverse kidney outcomes.

**Conclusions/interpretation:**

HSPC levels predict worsening of kidney function and improve the identification of individuals with type 2 diabetes and adverse kidney outcomes over and beyond a clinical risk score.

**Graphical Abstract:**

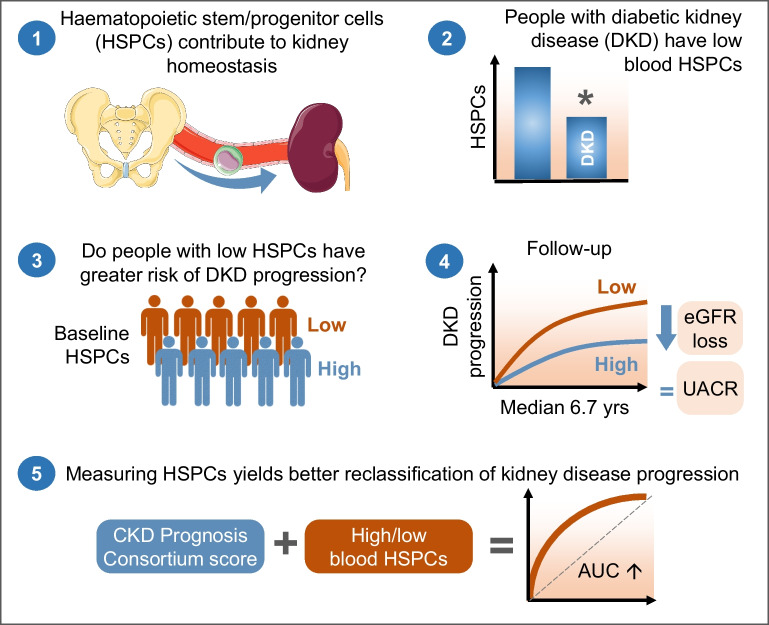


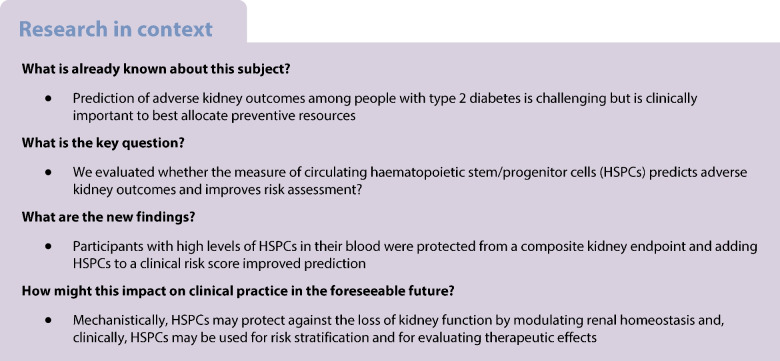


## Introduction

Type 2 diabetes is the most important cause of end-stage kidney disease (ESKD) in most countries in the world. Despite declining trends of mortality for CVD in the population with and without diabetes, mortality rates due to ESKD have remained stable over the last decades [1, 2].

Among individuals with type 2 diabetes, chronic kidney disease (CKD) results from a complex interaction of factors not limited to hyperglycaemia and including hypertension, obesity, chronic inflammation and hyperuricaemia, among others. As a consequence, the natural history of kidney disease often deviates from that of traditional diabetic nephropathy, and non-albuminuric CKD may prevail [3]. Notably, non-albuminuric CKD is characterised by an excess cardiovascular burden and shortened survival time [4].

Today, the unprecedented opportunity to prevent kidney disease with sodium–glucose cotransporter 2 (SGLT2) inhibitors mandates a thorough examination of an individual’s renal risk. Indeed, clinical trials show lower RR of adverse kidney outcomes with SGLT2 inhibitors across the spectrum of baseline kidney function but a lower absolute benefit in those with normal kidney function at baseline [5]. Therefore, CKD risk stratification could help resource allocation.

The mechanisms responsible for the onset of CKD, its progression and associated mortality among people with type 2 diabetes are incompletely defined [6]. The existence of a common pathway into which the various risk factors converge is fascinating. We and others have previously reported that individuals with type 2 diabetes display a consistent 30–40% reduction in the level of haematopoietic stem/progenitor cells (HSPCs) in the bloodstream [7]. This defect has been attributed to impaired structure and function of the bone marrow (BM), which physiologically releases HSPCs in a controlled way [8]. Notably, the occurrence of multiple risk factors that define the metabolic syndrome acts synergistically to reduce HSPC counts [9]. In different populations of individuals, including those with diabetes and the metabolic syndrome [10, 11], a low level of circulating HSPCs is strongly associated with increased rates of cardiovascular events, cardiovascular death and death from any cause [12]. While earlier studies used functional assays to demonstrate the correlation between circulating progenitor cells and vascular risk [13], recent studies have used flow cytometry to enumerate progenitor cells in the blood [14]. We have previously found that individuals with type 2 diabetes and below-median HSPC levels experienced a greater incidence of nephropathy over almost 4 years than did those with higher HSPC levels [15].

Herein, we evaluated whether HSPCs can predict the long-term occurrence of a broad spectrum of typical kidney outcomes of type 2 diabetes. Additionally, we examined whether adding the HSPC measure to a modern kidney risk assessment score improved patient stratification.

## Methods

### Participants

The study was conducted according to the principles of the Declaration of Helsinki. All participants provided written informed consent for HSPC analysis and for the re-use of clinical data for research purposes. According to national regulations on observational studies, the protocol was notified to and cleared by the Ethical Committee of the University Hospital of Padua (protocol no. 364n/AO/23).

We included individuals who underwent blood sampling for the quantification of circulating HSPCs at the Division of Metabolic Diseases of the University Hospital of Padua from January 2004 to April 2019. All participants were of white European ancestry. The same exclusion criteria applied throughout the study: active solid or haematological cancer; blood cytopenia (white blood cells <3000/μl; erythrocyte count <3,000,000/μl; platelet count <50,000/μl); acute inflammatory conditions; active autoimmune diseases or use of corticosteroids; advanced dementia; severe liver disease (cirrhosis Child–Pugh class B or C); ESKD (stage V); or inability to provide informed consent. For all participants, we recorded the following information: demographics (age, self-reported sex, diabetes duration); anthropometrics (height, weight, waist circumference); cardiovascular risk factors (obesity, smoking status, BP and diagnosis of hypertension, serum lipids and diagnosis of dyslipidaemia); diabetic complications; and current medications for the management of diabetes and other clinical conditions. Retinopathy was defined based on digital fundus examination. Nephropathy was defined in the presence of an eGFR <60 ml/min per 1.73 m^2^ or a urinary albumin/creatinine ratio (UACR) of 30 mg/g creatinine or higher. eGFR was calculated according to the CKD-EPI equation [16]. Neuropathy was defined based on symptoms and physical examination, and eventually confirmed by nerve conduction velocity or cardiovascular autonomic tests. Carotid atherosclerosis was diagnosed based on carotid ultrasound detection of ≥30% stenosis at one or more sites. Peripheral arterial disease was defined as claudication, rest pain or ischaemic wounds, with instrumental confirmation of lower-limb atherosclerosis. Heart failure was defined as the presence of a history of hospitalisation for heart failure or detection of an ejection fraction of less than 40%. Atrial fibrillation was diagnosed based on ECG findings. CHD was defined as a history of myocardial infarction, unstable angina or coronary revascularisation. Comorbidities, such as thyroid disease, gastrointestinal diseases and neurological diseases, were inferred from system-organ Anatomical Therapeutic Chemical (ATC) class medications H03, A02–07 and N03–07, respectively.

### HSPC analysis

The analysis of circulating HSPCs was performed on fresh peripheral blood samples, as previously described in detail [10]. Briefly, we labelled the cells with monoclonal antibodies against CD45 and the stem cell antigens CD34 and CD133. We treated the stained sample with a lyse-no-wash procedure (for erythrocyte lysis) and then analysed the cells by flow cytometry. HSPCs were defined as cells expressing the stem cell marker CD34 and/or CD133. Immature cell identity was confirmed by the CD45-diminished intensity. Given that CD45 and/or CD133 labelling were not always available, the minimal common definition of HSPCs consisted in the total number of CD34^+^ HSPCs. The absolute HSPC count was calculated by multiplying relative values (per 1,000,000 events) by the total white blood cell count (per μl of blood).

### Outcomes

Updated eGFR and UACR values were obtained by interrogating the participants’ electronic records from index date to the last observation, up to December 2022. The following kidney-specific outcomes were tested: new-onset macroalbuminuria (UACR of 300 mg/g or higher) confirmed in at least two separate measures; sustained eGFR decline of 30%, 40%, 57% or higher for at least 6 months; and ESKD (sustained eGFR <15 ml/min per 1.73 m^2^ for at least 6 months). Mortality was ascertained by accessing the hospital-based registry. Causes of death were categorised as cardiovascular, cancer, kidney or other causes, using definitions and procedures described in clinical trials [17]. The primary endpoint was a composite kidney outcome typically used in clinical trials [18], defined as the occurrence of any of new-onset macroalbuminuria, sustained ≥40% eGFR decline, ESKD or death from any cause. Secondary outcomes included components of the primary outcome (or alternative definitions of sustained eGFR reduction using 30% and 57% cut-offs), change in eGFR over time, annualised eGFR slope and change in UACR over time.

### Statistical analysis

Continuous variables are presented as mean and SD unless otherwise specified, when normally distributed. Variables that appeared to be non-normally distributed upon the univariate Kolmogorov–Smirnov test were log-transformed before being analysed with parametric tests and were presented as median and IQR. Categorical variables are presented as percentages. Participants were divided into two equal groups based on the median absolute level of circulating CD34^+^ HSPCs in the entire cohort. Comparisons between two groups were performed using the unpaired two-tailed Student’s *t* test for continuous variables or the χ^2^ square test for categorical variables. The Cox proportional hazards model was used to compare event-free survival rate for the primary endpoint and its components. The change over time in eGFR and UACR were compared using the mixed model for repeated measures (MMRM); time, group (high vs low HSPCs) and time-by-group interaction were entered as fixed factors and their effect was estimated. Autoregressive (first order) or compound symmetry was used as covariance structure. The annual eGFR slope was compared between groups using a univariate or multivariate linear regression analysis. All analyses were adjusted for the following predictors of kidney outcomes identified by the CKD Prognosis Consortium [19] as recorded at baseline: age; sex; BMI; systolic BP; HbA_1c_; UACR (log_*e*_); eGFR; atrial fibrillation; heart failure; CHD; and therapy with insulin, oral glucose-lowering medications and BP-lowering medications.

Performance in terms of renal outcome prediction was compared between model 1 (based on the risk score proposed by the CKD Prognosis Consortium [19]) and model 2 (risk score + high/low HSPC levels). Receiver operating characteristic (ROC) curves were constructed for all possible cut-offs for the two models and the area under the ROC curve (AUC or C statistic) was estimated and compared. The goodness-of-fit test by Hosmer and Lemeshow was used to evaluate calibration of the model. As alternative metrics to compare model performance, we also calculated the relative integrated discrimination improvement (rIDI) and the category-free net reclassification index (NRI) [20]. The analyses testing the improvement in predictive performance were performed using logistic regressions models as previously described [21]. The outcome of interest (composite of sustained decline in eGFR by 40% or more, or ESKD) was the dependent variable, whereas independent variables were the CKD Prognosis Consortium score and the HSPC status (high/low) or continuous variable (log_*e*_ scale)

SPSS Statistics version 28 or later (IBM, Armonk, NY, USA) or SAS software (version 9.4; SAS Institute, Cary, NC, USA), were used for statistical analyses and GraphPad Prism 5 (GraphPad Software, Boston, MA, USA) or later was used for drawing figures. Statistical significance was accepted at *p*<0.05.

## Results

### Participant characteristics

The study cohort was composed of 342 individuals whose baseline characteristics (Table 1) were representative of a contemporary population of patients with type 2 diabetes followed at diabetes outpatient clinics [22]. The mean age of the participants was 65.6 years, 67.8% were men, the mean diabetes duration was 13.4 years, and HbA_1c_ was 64 mmol/mol (8.0%). Of the whole cohort, 36.2% had either heart failure, coronary artery disease or peripheral arterial disease; 39.5% had baseline signs of nephropathy. About two-thirds (68.4%) of the participants were being treated with metformin and 38.3% were on insulin, with or without other glucose-lowering medications. The prevalence of participants receiving SGLT2 inhibitor or glucagon-like peptide-1 (GLP-1) receptor agonist treatment was low because recruitment largely occurred when such medications were not available. Participants received other appropriate therapies for the management of cardiovascular risk factors according to local practice at time of cohort entry.

**Table 1:** Baseline characteristics of participants

Characteristic	All (*N*=342)	Low HSPCs (*N*=171)	High HSPCs (*N*=171)	*p* value
Demographics and anthropometrics				
Age, years	65.6 (10.6)	67.8 (10.4)	63.4 (10.4)	<0.001
Male sex, %	67.8	63.7	71.9	0.106
Diabetes duration, years	13.4 (10.0)	13.0 (10.1)	13.8 (10.0)	0.470
BMI, kg/m^2^	29.3 (5.9)	28.7 (5.0)	29.9 (6.6)	0.045
Waist circumference, cm	101.2 (14.5)	99.5 (16.6)	102.9 (11.7)	0.028
Risk factors and laboratory results				
Current smoker, %	14.0	12.3	15.8	0.352
Former smoker, %	7.0	6.4	7.6	0.662
Systolic BP, mmHg	141.0 (18.9)	142.5 (19.3)	139.5 (18.5)	0.144
Diastolic BP, mmHg	72.5 (11.3)	71.3 (11.7)	73.7 (10.9)	0.053
Hypertension, %	84.5	88.9	80.1	0.025
HbA_1c_, mmol/mol	64 (13)	65 (14)	63 (10)	
HbA_1c_, %	8.0 (1.6)	8.1 (1.8)	7.9 (1.3)	0.173
Total cholesterol, mmol/l	4.4 (1.0)	4.3 (1.1)	4.4 (1.0)	0.660
HDL-cholesterol mmol/l	1.2 (0.4)	1.2 (0.4)	1.3 (0.4)	0.567
LDL-cholesterol, mmol/l	2.4 (0.9)	2.4 (0.9)	2.4 (0.8)	0.895
Triglycerides, mmol/l	1.6 (1.2)	1.6 (1.0)	142.0 (112.6)	0.649
Albuminuria, mg/g creatinine	123.8 (405.7)	182.3 (528.9)	1.6 (1.3)	0.008
Serum creatinine, µmol/l	87.4 (32.7)	88.4 (32.1)	86.4 (33.3)	
eGFR, ml/min per 1.73 m^2^	77.6 (20.4)	75.0 (21.0)	80.1 (19.4)	0.021
Complications, %				
Retinopathy	32.7	32.7	32.7	1.000
Nephropathy	39.5	45.0	33.9	0.036
Neuropathy	20.8	23.5	18.1	0.221
Atrial fibrillation	0.6	1.2	0.0	0.157
Heart failure	1.2	2.3	0.0	0.044
CHD	20.5	19.3	21.6	0.593
Peripheral arterial disease	24.3	28.1	20.5	0.102
Carotid atherosclerosis	56.1	57.9	54.4	0.515
Comorbidities, %				
Gastrointestinal disease	21.6	21.6	21.6	1.000
Thyroid disease	7.9	7.6	8.2	0.842
Central nervous system disease	8.2	8.2	8.2	1.000
Medications, %				
Metformin	68.4	66.1	70.8	0.353
Sulfonylurea	27.2	26.9	27.5	0.904
Glinides	3.8	3.5	4.1	0.778
Thiazolidinediones	3.2	1.2	5.3	0.032
SGLT2 inhibitors	11.1	10.0	12.3	0.912
GLP-1 receptor agonists	5.0	6.4	3.5	0.233
Insulin	38.3	38.6	38.0	0.912
RAS blockers	71.3	74.3	68.4	0.233
Other antihypertensive drugs	59.1	66.1	52.0	0.008
Statin	71.1	67.8	74.3	0.191
Antiplatelet agents	54.7	56.1	53.2	0.588
Uric acid-lowering drugs	8.8	7.0	10.5	0.253

Data are shown for the entire cohort and for the groups of participants divided by high/low levels (below-/above-median value) of circulating HSPCs

RAS, renin–angiotensin system

### Rates of kidney outcomes

**Fig. 1:**
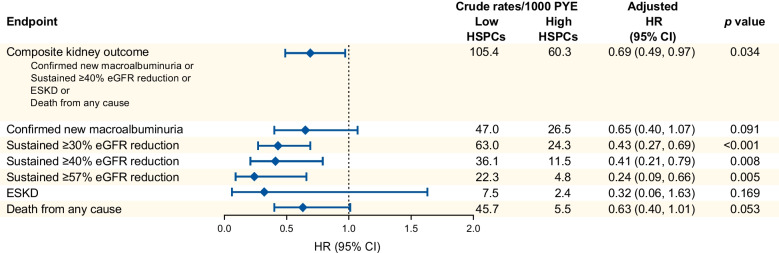
Forest plot of kidney outcomes by levels of circulating HSPCs. Participants were divided into two equal groups based on levels of circulating HSPCs: above-median (high); or below-median (low). For each combined and individual endpoint, the figure shows crude event rates/1000 person-years, the adjusted HR (95% CI) and the respective *p* values. PYE, person-years

Live status was available for 100% of participants, whereas updated eGFR and UACR were available for 98%. The median (IQR) follow-up time was 6.7 (4.1–10.0) years. The mean (SD) annual eGFR slope was −1.75 (5.2) ml/min per 1.73 m^2^, which is in line with the expected decline in a population at low-to-intermediate risk. The incidence rates (per 1000 person-years) of kidney outcomes are reported in Fig. 1. The most common outcome was a sustained ≥30% reduction in eGFR, followed by new-onset macroalbuminuria, whereas the occurrence of ESKD was a rare event. Eighty-eight participants died and causes of death were distributed as follows: cardiovascular 58.0%; cancer 21.6%; kidney 4.5%; and other 15.9%. The rate of the composite kidney endpoint was 81 per 1000 person-years (159 events in total).

The mean estimated 3 year risk of ≥40% eGFR decline or ESKD calculated using the equation derived from the CKD Prognosis Consortium [19], was 26%. In our cohort, 56 participants developed such an outcome, equal to a cumulative incidence of 16.6% and a rate of 24.3 per 1000 person-years. The risk estimated from baseline characteristics was notably higher and displayed a modest discrimination capacity against the observed outcome, with an AUC of 0.69 (95% CI 0.62, 0.76).

### HSPCs and kidney outcomes

Participants were divided into two equal groups (*n*=171 each) based on the median level of absolute CD34^+^ HSPCs (2428 cells/ml). As expected, the participants with low HSPCs were significantly older, had lower BMI and waist circumference, and a greater prevalence of hypertension, use of BP-lowering agents, heart failure and nephropathy (45.0% vs 33.9%; *p*=0.036), evidenced by lower eGFR and higher UACR at baseline (Table 1). The difference in the proportion of pioglitazone (a thiazolidinedione) users is consistent with the prior observation that pioglitazone can increase HSPCs [23]. HSPC analysis was repeated in 25 participants at a mean of 5.2 years from baseline: mean levels remained stable over time (baseline 2687 cells/ml; last observation 2589 cells/ml; *p*=0.84).

**Fig. 2:**
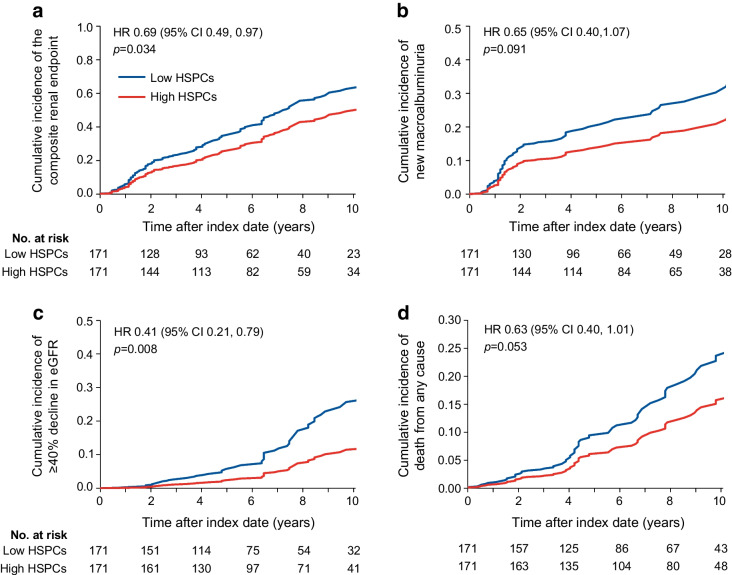
Incidence curves of selected kidney outcomes by HSPC levels. Participants were divided into two equal groups based on levels of circulating HSPCs: above-median (high); or below-median (low). Cumulative incidence curves and the numbers of participants at risk (No. at risk) are presented for the two groups. Adjusted HRs (95% CIs) from the Cox proportional hazard models are displayed along with the respective *p* values. (**a**) Composite kidney outcome (confirmed new-onset macroalbuminuria, sustained ≥40% eGFR decline, ESKD or death from any cause). (**b**) Confirmed new-onset macroalbuminuria. (**c**) Sustained decline in eGFR of 40% or more. (**d**) Death from any cause. The same population was used to compute occurrence of the components of the composite outcomes. Curves were truncated at 10 years because the number of participants at risk thereafter was very small. The number of ESKD events (*n*=12) was too small to be displayed

The primary composite kidney outcome (new-onset macroalbuminuria, sustained ≥40% eGFR reduction, ESKD or death), occurred significantly less often among participants with high HSPC levels as compared with those with low levels (adjusted HR 0.69 [95% CI 0.49, 0.97]; *p*=0.034; Fig. 2). This was mainly due to a lower rate of sustained eGFR decline, a finding that was confirmed when the endpoint was alternatively defined as ≥30% or ≥57% reduction (Fig. 1). The incidence of events of new-onset macroalbuminuria, ESKD and death from any cause was lower among participants with high HSPC levels but the difference was not significantly different between groups.

**Fig. 3:**
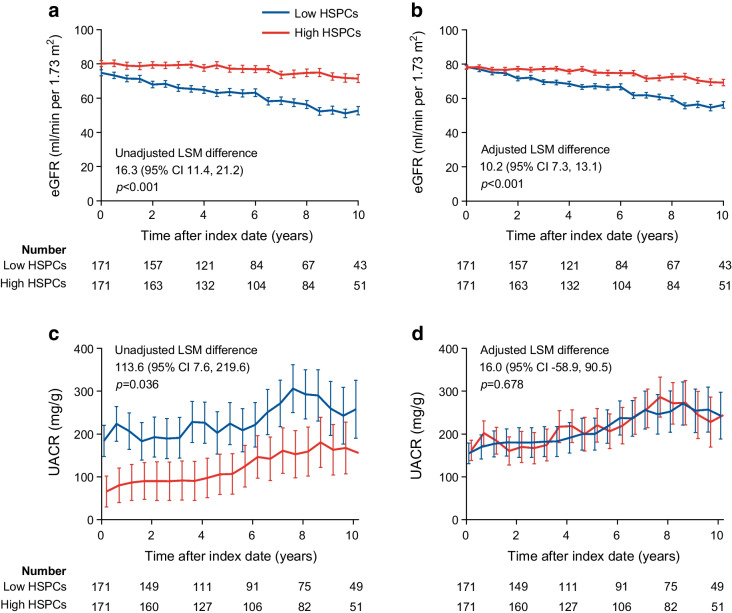
Change in eGFR and UACR by baseline HSPC levels. Participants were divided into two equal groups based on levels of circulating HSPCs: above-median (high); or below-median (low). (**a**, **b**) The change over time in eGFR, calculated using the MMRM, is shown for both groups before (**a**) and after (**b**) adjustment for covariates (predictors of kidney function loss in the CKD Prognosis Consortium risk score). (**c**, **d**) The change over time in the geometric mean of UACR is shown before (**c**) and after (**d**) adjustment for baseline albuminuria. Curves are truncated at 10 years and figures also report the numbers of participants at risk (No. at risk). The LSM difference between groups is shown along with the respective *p* values. Bars indicate SE

The observed annual eGFR slope was −3.4 and 0.01 ml/min per 1.73 m^2^ per year in the low and high HSPC groups, respectively (unadjusted *p*<0.001). After adjusting for predictors of kidney disease, the annual mean (SE) eGFR slope was 3.3 (0.6) ml/min per 1.73 m^2^ per year more negative in participants with low HSPCs (*p*<0.001). This is illustrated in Fig. 3a, which shows the changes over time in eGFR in the two groups. In the adjusted analysis (Fig. 3b), where the baseline difference in eGFR was abated, the least square mean (LSM) difference during follow-up was 10.2 ml/min per 1.73 m^2^ (*p*<0.001). For both the main kidney outcome and eGFR change, further adjustment for year of cohort entry did not modify results (not shown).

The UACR (geometric mean) remained significantly lower among participants with high HSPC levels from baseline to follow-up (Fig. 3c). When adjusted for the baseline value (log_*e*_), the change over time in UACR was similar between groups (Fig. 3d).

Visual inspection of the log_*e*_ (HSPCs) vs HR plot suggested a linear relationship without splines. The continuous level of HSPCs (per log unit) was significantly and independently associated with the composite outcome (HR 0.60 [95% CI 0.46, 0.78]), the loss of kidney function (HR 0.37 [95% CI 0.24, 0.57]) and all-cause mortality (HR 0.69 [95% CI 0.50, 0.97]).

### Discrimination of kidney outcome by HSPCs

**Fig. 4:**
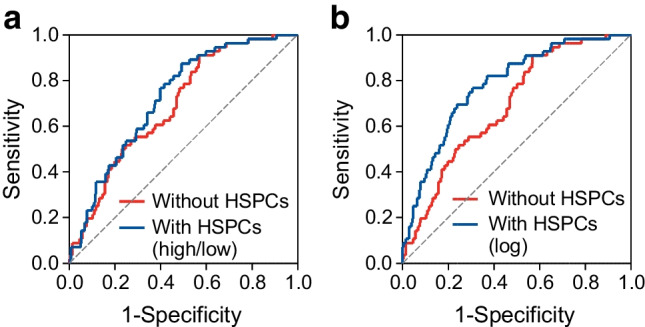
C statistics without and with HSPC levels. The C statistic was calculated against the composite outcome of ≥40% eGFR decline or ESKD using the risk score proposed by the CKD Prognosis Consortium without (red) or with (blue) the addition of high/low HSPC levels (**a**) or log_*e*_(HSPC count) (**b**). AUC (C statistics) significantly improved in both cases with HSPCs (see Results section). Analyses were conducted in the same set of participants with all data available for the two models

We then evaluated the usefulness of adding HSPC levels to the risk equation proposed by the CKD Prognosis Consortium to predict the composite outcome of ≥40% eGFR decline or ESKD [19]. As shown in Fig. 4a, when the high/low HSPC information was added to the risk score, we observed a significant increase in AUC from 0.695 to 0.751 (0.056 [95% CI 0.008, 0.104]; *p*=0.022). The model had a good calibration (Hosmer and Lemeshow goodness-of-fit *p*=0.7). Moreover, there was a remarkable and significant increase in rIDI (+30.6%; *p*=0.018) and an improvement in category-free NRI (NRI 0.59 [95% CI 0.33, 0.84]; *p*<0.0001) with 50% of events and 9% of non-events being correctly reclassified by the new model. There were 207 participants in so-called primary kidney prevention (i.e. with eGFR >60 ml/min per 1.73 m^2^ and UACR <30 mg/g). In these participants, discrimination by the CKD Prognosis Consortium [19] was not significant (AUC 0.564 [95% CI 0.415, 0.712]) but became significant after adding the high/low HSPC information (AUC 0.670 [95% CI 0.531, 0.808]).

When the HSPC variable was kept continuous and added to the CKD Prognosis Consortium score, AUC improved from 0.695 to 0.782 (Δ 0.088 [95% CI 0.016, 0.160]; *p*=0.017; Fig. 4b). rIDI improved by 74.9% (*p*=0.003) and category-free NRI improved by 0.56 (95% CI 0.28, 0.84; *p*=0.0001), with 21% of events and 35% of non-events being correctly reclassified with the new model.

## Discussion

Among people with type 2 diabetes observed for up to 10 years, a level of circulating HSPCs above the median value exerted a strong protective effect against the loss of kidney function and the future long-term occurrence of adverse kidney outcomes. Furthermore, adding the measure of HSPCs to the CKD Prognosis Consortium risk score [19] significantly improved the discrimination of individuals with a subsequent adverse kidney outcome.

These findings have mechanistic and clinical implications. Mechanistically, the traffic of HSPCs from the BM through the circulation may be involved in the homoeostasis of target organs of diabetic complications, including the kidney. In rodents, BM-derived cells reach the damaged kidney vasculature and participate in glomerular endothelial cell turnover [24]. Similarly, in individuals who received a sex-mismatched BM transplant, recipient cells contributed to turnover and regeneration of the kidney parenchyma, including tubular interstitial cells, as determined in kidney biopsies [25]. On this background, it is easy to understand why individuals with higher HSPC levels, reflecting a preserved BM cell traffic [7], were protected against adverse kidney outcomes. Furthermore, circulating cells homing to the diseased kidney may cooperate with resident kidney progenitor cells, which are activated after injury, and share some antigenic features of HSPCs [26].

High HSPC levels were associated with a slower decline in eGFR but not with a reduction in the worsening of albuminuria. Thus, we hypothesise that HSPC levels may be linked to non-albuminuric CKD, a condition characterised by excess cardiovascular burden and high mortality [4]. Consistently, there is a close connection between HSPCs and various cardiovascular risk factors that contribute to non-albuminuric CKD, including obesity, hypertension and hyperuricaemia [23, 27, 28].

On the other hand, elevation in albuminuria may precede the decline in HSPC levels evidenced by the higher UACR in individuals with low HSPC levels. Those with higher levels of HSPCs may have a less severe remodelling of the BM niche [7, 29] because the release of HSPCs into the bloodstream relies on the functional integrity of the BM microcirculation. Diabetes features rarefaction and increased permeability of the BM microvasculature [30], exposing HSPCs to pro-oxidant conditions [31]. Notably, in people with type 2 diabetes, raised albuminuria is a sign of generalised vascular dysfunction not limited to glomerulopathy [32]. Therefore, the association between high albuminuria and low circulating HSPCs over several years may be a proxy of BM microvascular remodelling and hyperpermeability. In this view, reduction in HSPC levels could be considered one of the systemic consequences of generalised endothelial dysfunction and vascular permeability, explaining part of the negative prognostic impact of albuminuria on cardio-renal endpoints.

Clinically, we show that knowing an individual’s HSPC level can improve the prediction of kidney function loss based on routinely available information. As CKD remains largely undiagnosed until its later stages [33, 34], identifying strategies for better risk stratification is highly relevant. This is particularly true as new treatments can prevent CKD development or progression (e.g. SGLT2 inhibitors, GLP-1 receptor agonists and non-steroidal mineralocorticoid receptor antagonists) [35]. In clinical trials and observational studies, use of SGLT2 inhibitors was associated with a remarkable protection against new CKD, even in primary prevention (i.e. in individuals with normal eGFR or albuminuria) [5, 36]. Thus, in the absence of early predictors of CKD, treatment with SGLT2 inhibitors should be considered for most patients with type 2 diabetes. Yet, the global burden of diabetes mandates that interventions are sustainable. Thus, improving the prediction of CKD may help better allocation of expensive preventive strategies. The score developed by the CKD Prognosis Consortium [19] uses readily available clinical variables but has shown suboptimal performance, especially when applied to independent external populations, leaving space for improvement. We recognise that the HSPC measure may not be readily available in primary care and in some outpatient settings. In addition, a similar extent of prediction improvement might be achieved by other laboratory measures [37]. As a proof-of-concept, it is fascinating that HSPCs grant at the same time a protective biological effect and an improved prediction against kidney outcomes. It is intriguing that SGLT2 inhibitors improve HSPC levels and BM-derived cell traffic to sites of vascular damage [38–40]. That modulation of HSPCs mediates at least part of the nephron-protective effect of SGLT2 inhibitors is a fascinating hypothesis.

We acknowledge other study limitations. Despite a long follow-up, sample size was relatively small, affecting the analysis of rarer outcomes (e.g. ESKD or cause-specific mortality). The need to analyse HSPCs in fresh blood samples hampers the possibility of performing larger studies and the availability of external cohorts for replication. In addition, our cohort was recruited over a period of 15 years, during which time the standards of care for the management of diabetes, cardiovascular and renal risk have changed dramatically. Similarly, though we kept the protocol for HSPC enumeration the same from 2003 onwards, variations in reagent lots and instrumental calibration may have occurred. Although such a degree of heterogeneity may have diluted the strength of the associations, adjustment for cohort entry year did not modify the results.

In summary, we found that higher HSPC levels in type 2 diabetes protects against the loss of kidney function in the long term. Whether drugs like SGLT2 inhibitors may act through this pathway is unknown but we argue that strategies to preserve BM function and HSPC levels might improve kidney outcomes in type 2 diabetes.

## Data Availability

Restrictions apply to the availability of source data used for this study, mainly for privacy policy. The datasets generated during the current study are not publicly available but may be available from the corresponding author upon reasonable request.

## Abbreviations

BM: Bone marrow
CKD: Chronic kidney disease
ESKD: End-stage kidney disease
GLP-1: Glucagon-like peptide-1
HSPC: Haematopoietic stem/progenitor cell
LSM: Least square mean
MMRM: Mixed model for repeated measures
NRI: Net reclassification index
rIDI: Relative integrated discrimination improvement
ROC: Receiver operating characteristic
SGLT2: Sodium–glucose cotransporter 2
UACR: Urinary albumin/creatinine ratio

## Authors’ relationships and activities

BMB received lecture or advisory board fees from Astra Zeneca, Boehringer Ingelheim, Eli Lilly, Mundipharma, Novartis, Novo Nordisk and Sanofi. MLM received lecture fees or advisory board fees from Eli Lilly, MSD, Novo Nordisk, SLA-Pharma and Servier. AA received research grants and lecture or advisory board fees from MSD, AstraZeneca, Novartis, Boehringer Ingelheim, Sanofi, Mediolanum, Janssen, Novo Nordisk, Lilly, Servier and Takeda. GPF received grants, honoraria or lecture fees from Abbott, AstraZeneca, Boehringer, Lilly, MSD, Novo Nordisk, Sanofi, Servier and Takeda. The other authors declare that there are no relationships or activities that might bias, or be perceived to bias, their work.
